# A Four-Step Method for the Development of an ADHD-VR Digital Game Diagnostic Tool Prototype for Children Using a DL Model

**DOI:** 10.3389/fpsyt.2020.00829

**Published:** 2020-08-17

**Authors:** Tjhin Wiguna, Ngurah Agung Wigantara, Raden Irawati Ismail, Fransiska Kaligis, Kusuma Minayati, Raymond Bahana, Bayu Dirgantoro

**Affiliations:** ^1^ Faculty of Medicine, Universitas Indonesia—Dr. Cipto Mangunkusumo General Hospital, Jakarta, Indonesia; ^2^ Faculty of Computer Science, Bina Nusantara University, Jakarta, Indonesia

**Keywords:** attention-deficit/hyperactivity disorder, virtual reality, machine learning, diagnostic tool, neuropsychological test, Indonesia, digital game

## Abstract

Attention-deficit/hyperactivity disorder (ADHD) is a common neurodevelopmental disorder among children resulting in disturbances in their daily functioning. Virtual reality (VR) and machine learning technologies, such as deep learning (DL) application, are promising diagnostic tools for ADHD in the near future because VR provides stimuli to replace real stimuli and recreate experiences with high realism. It also creates a playful virtual environment and reduces stress in children. The DL model is a subset of machine learning that can transform input and output data into diagnostic values using convolutional neural network systems. By using a sensitive and specific ADHD-VR diagnostic tool prototype for children with DL model, ADHD can be diagnosed more easily and accurately, especially in places with few mental health resources or where tele-consultation is possible. To date, several virtual reality-continuous performance test (VR-CPT) diagnostic tools have been developed for ADHD; however, they do not include a machine learning or deep learning application. A diagnostic tool development study needs a trustworthy and applicable study design and conduct to ensure the completeness and transparency of the report of the accuracy of the diagnostic tool. The proposed four-step method is a mixed-method research design that combines qualitative and quantitative approaches to reduce bias and collect essential information to ensure the trustworthiness and relevance of the study findings. Therefore, this study aimed to present a brief review of a ADHD-VR digital game diagnostic tool prototype with a DL model for children and the proposed four-step method for its development.

## Introduction

Attention-deficit/hyperactivity disorder (ADHD) is a disorder characterized by excessive hyperactivity and greater impulsive and inattentive behavior than in peers of the same age group, resulting in disturbances in the patients’ daily functioning, such as studying and interacting with the environment. Moreover, children with ADHD are known to be targets of bullying and become scapegoats for several unwanted situations, which may eventually cause feelings of isolation and trigger other mental disorders, such as behavior disorders, mood disorders, or Internet addiction disorders, in the future (1). Previous studies have found that the prevalence rate of ADHD ranges from 3–15%, making this condition the most common mental disorder among children of elementary school age group (1, 2). In Indonesia, the national prevalence of ADHD in the elementary school age group is currently unavailable. However, a study by Suryani et al. (3) found that nearly 26% of the children from first to sixth grades were diagnosed with ADHD at 27 elementary schools selected randomly in Jakarta (3).

In the Diagnostic and Statistical Manual of Mental Disorders, Fifth Edition (DSM-5), ADHD is categorized as a neurodevelopmental disorder highlighted by deviations of behavior and emotional, cognitive, and psychosocial development (4). Studies have shown that the psychopathologies of ADHD are related to executive dysfunction, resulting in children’s inability to control their emotion and behavior. Moreover, studies using fMRI BOLD, PET, or SPECT have also revealed less activity in the dorsolateral prefrontal cortex (DLPFC) in children with ADHD (5–7). Another study related to genetic evaluation found that ADHD was associated with dopamine and nor-epinephrine transporter gene polymorphism (8–10).

Chiatric interviews, observation of the child, and interviewing children and parents based on the diagnostic criteria of the DSM-5 or International Classification of Diseases-10/11 (ICD-10/11). During the mental state examination, several symptoms might or might not be exhibited, depending on the children’s familiarity with and adaptability to the interview and observation setting. Thus, the results of psychiatric examinations to establish diagnosis of ADHD mostly come from the parents. Furthermore, ADHD can co-exist with other neurodevelopmental disorders, such as intellectual disability, autism spectrum disorder, or other specific learning disability and mood disorder like depression or pediatric bipolar disorder. Therefore, it is necessary to develop an ADHD diagnostic tool that is highly sensitive and specific to ADHD clinical symptoms, not only to justify the ADHD diagnosis but also to create a non-threatening environment for children. Several studies have shown that virtual reality (VR) can be used as a diagnostic tool for ADHD because of its ability to create a virtual environment that is more enjoyable and able to collect better behavioral, inattention, and distractibility data than ordinary neuropsychological tests (11, 12). Most studies used continuous performance tests (CPTs) or other neuropsychological tests for standardized testing (13–15). In addition, the ADHD-VR diagnostic tool development did not include a machine learning application (artificial intelligence application) enabling computer systems to automatically learn and improve from experience without being explicitly programmed. Machine learning is capable of receiving some data (input data), training a model on the data, and finally using the trained model to make predictions on new data (output data) that have more accurate diagnostic value (16).

Due to advancements in technology, diagnostic tools for children with ADHD that is sensitive and specific to ADHD clinical symptoms can be improved by merging a child’s needs and modern technologies, such as VR, with a deep learning (DL) model. In recent years, DL has become quite valuable in the machine learning used in many routine applications, such as VR digital games. DL is a subcategory of machine learning that processes data through many layers of artificial neurons (convolutional neural networks/ConvNets) between the input and output data. These processes are said to be similar to those of the human brain (17). When such networks receive multiple data, their neurons individually respond within each layer (e.g., convolution) to define the data into a single value significant for diagnostic purposes. Therefore, all the data pass through each layer in the ConvNets sequentially until the input and output data can be used to generate final judgment forms. DL has become increasingly relevant in many fields of artificial intelligence besides VR digital games (18). Thus, a DL model can be applied to develop a more accurate ADHD-VR digital game diagnostic tool that is not only sensitive and specific to ADHD clinical symptoms but also creates a playful experience. In addition, the ADHD-VR digital game diagnostic tool prototype for children using a DL model can be of benefit in places where mental health resources are low, as it can provide diagnostic capabilities in standard health services or tele-psychiatry consultation and thus permit the delivery of management recommendations. Therefore, this study presents a brief discussion of an ADHD-VR digital game diagnostic tool prototype for children using a DL model and the proposed four-step method for its development to improve the completeness and transparency of reports of diagnostic tool accuracy.

## An ADHD-VR Digital Game Diagnostic Tool Prototype for Children Using a DL Model

Diagnostic tools are essential in modern medical practices in child and adolescent psychiatry, as they can be used to confirm a diagnosis, provide more evidence to eliminate one diagnosis versus another, provide more evidence for parents, or rule out a differential diagnostic. Although psychiatric examination or other self-rating questionnaires are the main approaches used in ADHD diagnostic procedures, laboratory testing or imaging examinations are used as well, especially to rule out a general medical condition or to identify any medical conditions that mimic ADHD. Nowadays, advancements in biological psychiatry have suggested several hypotheses based on the biological markers for ADHD; however, this area of investigation is still developing. Meanwhile, although there is no device that offers the desired diagnostic accuracy for ADHD clinical symptoms, several devices have been developed to investigate the neuropsychology and electrophysiology of the brain or to perform imaging examinations (19–22).

Nevertheless, the concept of games for health is another important area that should be considered. Under this concept, a game is a system where players engage in conflicts that they need to solve. Hence, a game can be enjoyed by children because it is challenging, consists of rules that stimulate creativity, contains specific goals that need to be completed by the players, provides a feedback system telling them how are they doing, and yields quantifiable outcomes (23, 24). VR digital games based on a DL model are one of the best approaches; it could be used as a source of data that can yield diagnostic value in DL technology. In addition, VR digital games create a playful environment with stimuli that are not directly confrontational and recreate experiences that would be impossible in the real world with high realism (25). Thus, VR digital games with a DL model are a promising diagnostic tool for children with ADHD in the near future.

In the last decade, new model of CPTs have been developed using VR technology. VR, one of the most cutting-edge technologies used in the computer science field, was originally developed by Ivan Sutherland and colleagues in 1960 and has grown rapidly since the last decade. Since its beginnings and translations into various areas, several definitions have been proposed for VR, such as real-time interactive graphics with 3D models combined with a display technology that immerses the user in the model world while allowing its direct manipulation. On the other hand, VR has also been defined as the illusion of participation in a synthetic environment rather than external observation of such an environment. VR relies on three-dimensional, stereoscopic head-tracker displays, hand/body tracking, and binaural sound. VR is an immersive, multi-sensory experience. Lastly, VR was described by Cruz-Neira in 1993 as “Virtual reality refers to immersive, interactive, multi-sensory, viewer-centered, 3D computer generated environments and the combination of such technologies that are required to build the objective of a virtual world” (25, 26).

Compared to other media, VR requires greatly heightened immersive techniques; they increase productivity and retention and are easily understood, especially for coding, development, and training. Similarly, VR enables greater spontaneity in users, which strengthens the effect of interactivity. Therefore, VR can be used to perform tasks more easily and with greater comprehension. There are two core components of VR functional aspect: immersion and interactivity. Furthermore, use of a DL model for machine learning can allow more vivid VR experiences as diagnostic tools with more accurate prediction (17). The experiences of a VR user can be uncovered by measuring the presence, realism, and level of reality. Presence is defined as a complex mental feeling of “being there” that involves the sensation and perception of physical awareness as well as the likelihood of interacting and reacting as if the child were in the real world. Equivalently, the realism levels correlate to the degree of presumption that the child has about the real experience. For instance, if the appearance of VR is similar to the real world, the VR user’s expectations will be those for the presumption of reality, thereby enhancing the experience inside the VR world. Similarly, the higher the level of reality in connection with the virtual improvement, the higher the authenticity of the VR user’s experience and the more accurate the prediction of diagnostic results (25, 26).

An ADHD-VR digital game diagnostic tool prototype using a DL model can be designed simplistically with minimal distractions. For example, the child can control virtual hands to grab, release, throw, and place objects. Furthermore, the child can point a laser from his/her index finger for item selection. The environment can be also limited, limiting physical movement, so that children only need a small physical space to operate the VR digital game, allowing them to perform actions while standing or sitting. The more minimized (calm and easy environment) the VR, the less motion sickness they might experience, helping the child maintain a constant reference to the activity. Furthermore, a VR digital game with a DL model interface would surround the child with various tools having diagnostic value. Children can grab layers from a toolbox to their left or right and place them within a “working space,” a defined region in front of the child containing several sequences of layers within which children can insert, remove, and rearrange layers. After the child grabs a layer from the toolbox, a new layer of the same type will spawn to take its place. In this manner, more than one layer of a given type can be present within a DL model. The child can define the toolbox from left to right and train it by pressing or selecting a button to the left or right of the “working space”. These operations allow for the simple construction and modification of a functional ConvNet. While computing, a display in front of the child reports the status of playing (17, 27). Upon completion, the display reports the accuracy of the diagnosis from the results of DL computed against a standardized testing set; in this way, the ADHD clinical symptoms are based on DSM 5 or ICD 10/11 or other ADHD standardized symptom scales. Therefore, the input and output data that come from a VR digital game using a DL model are much more precisely transformed into a single value for diagnostic purposes. Therefore, VR digital games using a DL model will likely be at the forefront of diagnostic tools for ADHD in the coming years.

In recent years, several studies of ADHD-VR diagnostic tools have been conducted showing that VR is a promising diagnostic tool for ADHD despite not including machine learning or DL applications, and CPT could be of benefit as standardized testing. Neguț et al. (13) developed a classroom VR-CPT (inside the classroom) with ADHD children as the research participants; however, the research only focused on the inattention domain, and the results revealed that the children with ADHD needed more time to complete the response task of VR than conventional CPT, and the difference was statistically significant. Diaz-Oruega et al. (28) developed the AULA Virtual Reality Continuous Performance Test (AULA VR-CPT) to measure inattention in children with ADHD compared to conventional CPT; the result revealed a positive correlation between the AULA VR-CPT and conventional CPT. Moreover, Diaz-Oruega’s study also found the AULA VR-CPT to be capable of distinguishing between attention inability among children with ADHD who were taking medication and those who were not in terms of attention, impulsivity, and motoric activity. Another study by Fang et al. (15) applied discriminant analysis of the Virtual Reality Medical Center system (VRMC) of correct items, incorrect items, total time, and accuracy to show that they were significantly associated with the levels of impulsion/hyperactivity and ADHD index as measured by Conners’ Parent Rating Scale (CPRS) and CPT. Aceres et al. (14) presented a VR-CPT test (Nesphola Aquarium) for adolescents and adults that was designed to measure executive function. The study used the Adult ADHD Self-Report Scale (ASRS) as the ADHD symptom scale. The results showed that the VR test Nesphola Aquarium significantly predicted current and retrospective ADHD symptoms (14). A meta-analysis study by Parsons et al. (29) concluded that virtual reality classroom CPT was effective in assessing attention performance in children with ADHD. Moreover, it could also differentiate attention differences between children with ADHD and typically developing children; however, it was unclear whether the differences were directly related to the VR classroom environment or other factors. Thus, there is a need for better designed and adequately powered studies to investigate VR, especially with machine learning as a diagnostic tool for children with ADHD.

## Discussion and Future Direction: The Proposed Four-Step Method for ADHD-VR Digital Game Diagnostic Tool Prototype for Children With DL Model Development

Diagnostic tool development is at risk of bias, especially with respect to accuracy. Generally, major sources of bias originate in unclear methodological design, such as research subject recruitment, data collection, the execution or interpretation of the test, or data analysis. As a result, the estimates of sensitivity and specificity of the tool being compared against the reference standard can be flawed, deviating systematically from what would be obtained under ideal circumstances (30). Biased results can lead to improper recommendations about tools, negatively affecting patient outcomes or healthcare services. Diagnostic tool accuracy is not a fixed asset of a tool. A tool’s accuracy in identifying patient diagnosis typically varies between settings and patient groups and depends on prior testing (31). These sources of variation in diagnostic tool accuracy are relevant for ADHD-VR digital game diagnostic tool prototypes for children using DL model development because the diagnostic tool may be used in hospital settings, primary care settings, or school settings to diagnose ADHD. Consequently, the risk of bias and concerns about applicability are the two key factors that a diagnostic tool needs to address to minimize design-related bias in studies (30, 32, 33).

Therefore, the proposed four-step method is meant to reduce these biases and collect essential information, including the study design and research subjects, to judge the trustworthiness and relevance of the study findings. Furthermore, the proposed four-step method also serves as a mixed-method study that combines qualitative and quantitative methods to aid the development of diagnostic tools in psychiatry (34). The strengths that can be generated from the proposed four-step method for ADHD-VR diagnostic tool prototypes for children using a DL model are, first, that it guides a systematized approach to diagnostic tool development, confirming that the research findings can be translated into clinical utility in a timely manner. Second, it could prevent premature dissemination of the findings into the extensive clinical use. Both of the above strengths serve to ensure a balance between patient care needs and economic concerns.

In first step, the qualitative part uses the Delphi technique with a focus group discussion (FGD) method to collect information to build a theoretical concept of an ADHD-VR digital game diagnostic tool for children using a DL model. The FGD would include several mental health professionals, such as child psychiatrists, psychiatrists, developmental psychologists, teachers, parents, computer science experts, and game designers. The first round of the FGD will discuss the clinical symptoms of ADHD based on the DSM 5, ICD-10/11, and their own perspectives. The next discussion would concern how to translate these symptoms into the VR digital game and use machine learning or a DL conceptual framework to transform the input and output data into a single ADHD diagnostic value to support ADHD diagnosis. The second round of the FGD will discuss the concept of a VR digital game diagnostic tool prototype for children using a DL model based on the results of the previous round. The first and second round of FGD may be conducted several times to reach agreement within the group. In addition, the agreement provides a replication concept by either the same or different collaborating groups. From the first and second FGD results, computer science experts and VR digital game designers can begin work to establish the prototype of an ADHD-VR digital game diagnostic tool using a DL model. In this step, the third-round FGD (final round) must include other independent experts and children under the age of 12 years. In this final round, the goals are not only to reach agreement on an ADHD-VR digital game diagnostic tool prototype using a DL model for children but also to confirm whether the diagnostic tool is suitable for use with children. In this way, content validation is achieved.

The second step is a feasibility study to lay the foundation of the plan and to reduce or eliminate problems that limit the successful delivery of the accuracy study of the ADHD-VR digital game diagnostic tool prototype using a DL model in a clinical setting (the third step). Based on the United Kingdom’s National Institute for Health Research Evaluation, Trials and Studies Coordination Centre (35), a feasibility study is the stage of research before the third step of a study in order to answer the question, “Can a diagnostic tool accuracy study be performed?” area feasibility study is conducted to estimate important parameters needed to design the main study; a feasibility study does not evaluate the outcome of interest. Moreover, it is not an analytical study designed to test a hypothesis. Instead, it is usually focused on gathering data for use in optimizing diagnostic tool modifications (36). The feasibility study is generally an open-label study (for example, it does not include a control group) and is conducted at a single site with fewer than 25 children with ADHD. In this step, research subjects are asked to try out the ADHD-VR digital game diagnostic tool prototype and report their experiences individually as feedback to improve and modify the diagnostic tool. Meanwhile, the input and output data of ADHD-VR digital game diagnostic tool prototype can be generated into the DL model to finally yield diagnostic value. The more the data generated into the DL model, the more accurate the diagnostic result would be. The results of this step are used to evaluate the ADHD-VR digital game diagnostic tool prototype for children with ADHD, and it is necessary to make changes to arrive at the feasible and accurate diagnostic tool to be tested in the next step.

The third step is a diagnostic tool accuracy study (a validity and reliability study) to identify the performance of the ADHD-VR digital game diagnostic tool prototype using a DL model for children. In this step, the results are sensitivity, specificity, likelihood ratio, and positive and negative predictive values. Therefore, the research subjects are children with ADHD and a control group of healthy children; if possible, comparisons can be made with other children whose diagnoses commonly mimic ADHD clinical symptoms, such as autism spectrum disorder, intellectual disability, disruptive behavior disorder, and depressive disorder. The gold standard or standardized testing is the ADHD diagnosis based on the agreement of a number of experts using an ADHD standardized clinical symptom scale or standardized psychiatric examination guidelines for ADHD based on DSM 5 or ICD-10/11 criteria. Although the nature of the behavioral and emotional symptoms of ADHD is heterogeneous, it would be expected that the ADHD-VR digital game diagnostic tool with DL model would be sensitive and specific enough to identify the respective patterns and produce an accurate diagnostic value. Therefore, the number of the research subjects should be adequate for these purposes. In a diagnostic study, the pre-determined value for both sensitivity and specificity must be at least 0.70, and it must be indicative that the probability of the tool detecting a true-positive or a true-negative is at least 70%. Based on the recommendations by Bujang and Adnan (37), if we estimate the ADHD prevalence in Indonesia at 10%, the total number of research subjects in this step would be 310 children, including 31 children with ADHD, to achieve a minimum accuracy of 80.7% so as to detect a change in the sensitivity from 0.70 to 0.90, where a statistically significant level is 0.048. The minimum sample size adequate to detect a change in the value of specificity from 0.70 to 0.90 is 34, including 3 children with ADHD. Furthermore, other important issues that must be considered in this step are defining the clinical characteristics of the ADHD sub-types identifiable by psychiatric examination guidelines or ADHD standardized scales, the duration of ADHD, the severity of the illness, and medication effects.

The fourth step is multicenter clinical trials of the ADHD-VR digital game diagnostic tool prototype for children using a DL model. Multicenter clinical trials should pave the way toward standardization of the diagnostic tool procedures for cost effectiveness and impact on both short-term and long-term clinical uses. While the earlier steps included smaller samples of comparison subjects that are usually locally formed, Step 4 needs to develop larger normative databases that can eventually be used to examine an individual’s data. The development of such databases can be challenging and will require collaboration among research groups concerned with the specific tool that is being developed. The results of this step will be used to re-evaluate the usefulness of the diagnostic tool prototype before it is generalized for clinical use.

There are several issues that still need to be carefully examined whenever applying the proposed four-step method diagnostic tool development, such as the fact that qualitative information can be judgmental and biased; therefore, the qualitative data should be collected from many sources until it is condensed. Moreover, the proposed four-step method is originally designed for mixed-method studies. Thus, it needs a careful plan especially to describe all aspects of research, including the research subjects for the qualitative and quantitative parts; the timing (the sequence of qualitative and quantitative parts); and the plan for integrating data. However, it affords us the opportunity to ensure that the study findings are grounded in participants’ experiences, providing more and more varied information than can be obtained from strictly quantitative research, and is able to support multidisciplinary team research by encouraging the interaction of quantitative, qualitative, and mixed-methods scholars.

## Conclusion

ADHD is one of the prevalent neurodevelopmental disorders in a clinical setting. VR and machine learning, such as DL technologies, hold great promise for application in human health diagnostic tools in the near future, especially in areas with low health and mental health resources, such as general practitioners, child psychiatrists, general psychiatrists, psychologists, and behavior pediatricians. Therefore, by utilizing an ADHD-VR digital game diagnostic tool prototype for children with a DL model in standard health services or tele-psychiatry consultation, ADHD can be diagnosed and early management can be delivered, thereby reducing the impact of the illness. In addition, it can provide parents with much clearer evidence for an ADHD diagnosis. In addition, the proposed four-step method, which is based on mixed-method research concepts, holds promise as an approach to develop an optimal diagnostic tool because it is designed to improve the completeness and transparency of reports of diagnostic tool accuracy studies.

## Data Availability Statement

The datasets generated for this study are available on request to the corresponding author.

## Ethics Statement

The studies involving human participants were reviewed and approved by The Ethic Committee of the Faculty of Medicine University of Indonesia, number: KET-503/UN2.FK/Etik/PPM.00.02/2019. Written informed consent to participate in this study was provided by the participants’ legal guardian/next of kin.

## Author Contributions

TW contributed to the design of the manuscript, literature review, manuscript draft and preparation, and writing the manuscript. NW contributed to the literature review and manuscript preparation. RI, FK, RB, and BD conceptualized and reviewed the manuscript.

## Funding

This paper was funded by a “Hibah Q1Q2” grant from Universitas Indonesia Research Funding 2019. The funder had no role in study design, data collection, and data analysis, decision to publish, or manuscript preparation.

## Conflict of Interest

The authors declare that the research was conducted in the absence of any commercial or financial relationships that could be construed as a potential conflict of interest.
